# Supplementary material for: Species-Specific Responses to Human Trampling Indicate Alpine Plant Size Is More Sensitive than Reproduction to Disturbance
Source: Plants (Basel). 2023 Aug 24;12(17):3040. doi: 10.3390/plants12173040 (PMC10489881; doi:10.3390/plants12173040)
Supplement: Supplementary file 1 [file plants-12-03040-s001.zip › plants-2506383-supplementary.pdf]

SUPPLEMENTARY INFORMATION

Table S1. Model fitting details. Model fitting details for each species traits model, including the model family, number of iterations, Bulk Estimated Sampling Size (ESS), ability of the model to appropriately fit data skew, number of observations with a Pareto K value > 0.7, and how well dispersion was modeled. We set ‘init = 0’ for plant percent cover and reproduction models to aid the model in finding initial values. Main results are shown in Table 1.

Species	Trait	Family	Iterations	Bulk ESS	Skew	Pareto K > 0.7	Dispersion
[All plants]	Percent Cover	Beta	5000	3727	poor	0	good
P. empetriformis	Height	Negative Binomial	5000	3631	very good	0	moderate
	Diameter	Negative Binomial	5000	2263	moderate	0	very good
	Reproduction	Beta	5000	12748	very poor	1	poor
C. mertensiana	Height	Negative Binomial	5000	4367	very good	0	good
	Diameter	Negative Binomial	5000	3436	poor	0	very good
	Reproduction	Beta	5000	6233	very poor	1	poor
V. ovalifolium	Height	Negative Binomial	5000	4568	poor	1	moderate
	Diameter	Negative Binomial	5000	5097	poor	1	very good
	Reproduction	Beta	5000	10017	very poor	1	poor
Carex spp.	Height	Negative Binomial	5000	6113	good	1	moderate
	Diameter	Negative Binomial	5000	5617	moderate	1	very good

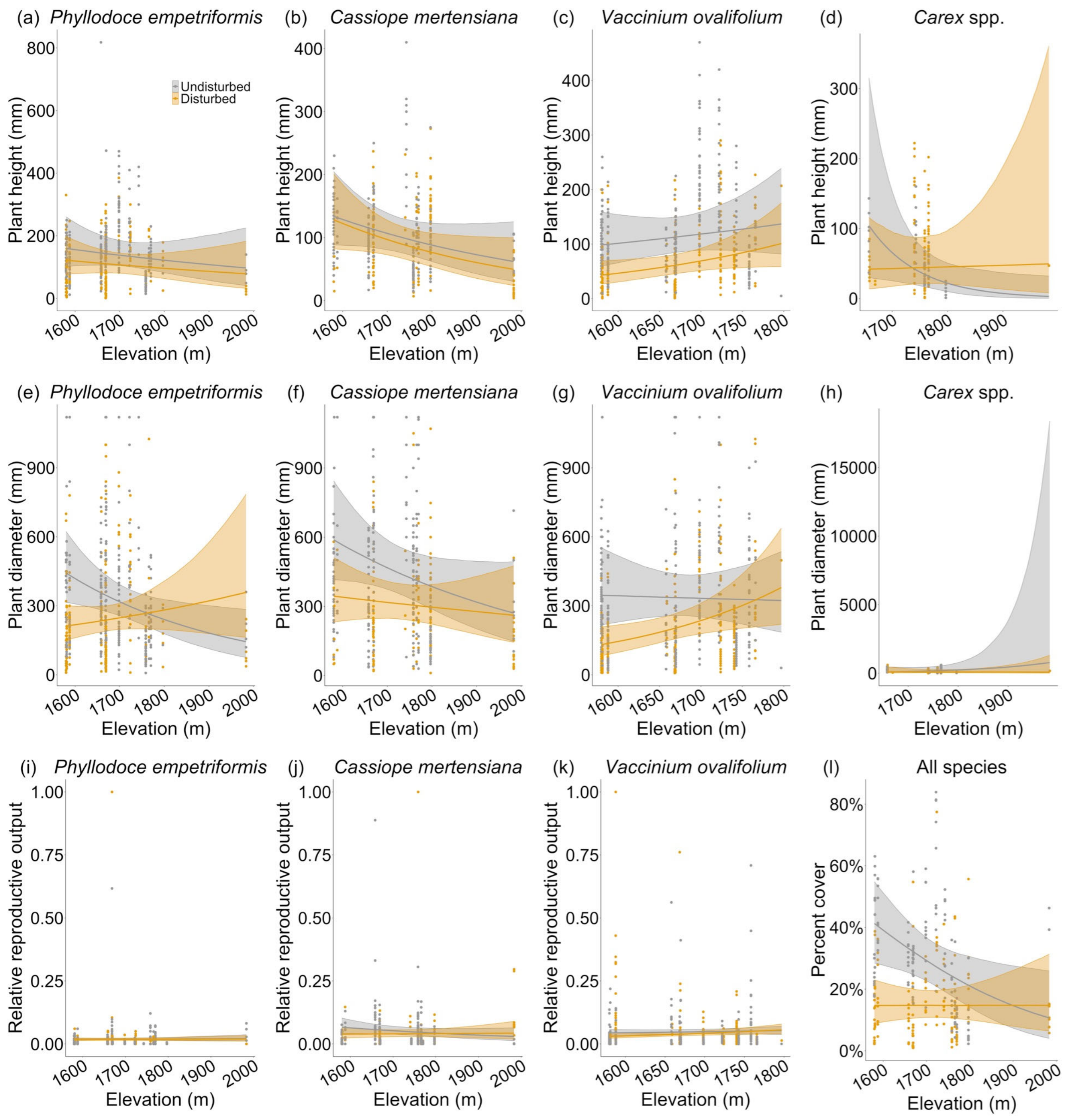

Figure S1. Effects of disturbance and elevation. Elevation does not affect plant maximum height, maximum diameter, reproductive output (summed buds, flowers, fruits by total plant area, relative to the maximum per species) or plant percent cover. Disturbance effects only increase with elevation for *Carex* spp height. Shown are parameter estimates of elevation (lines) with their credible intervals (shading) from Bayesian hierarchical non-linear mixed models. Parameter estimates of disturbance only are shown in Fig. 2. Legend for all plots is as in **(a)**. Note that y-axes are on different scales.

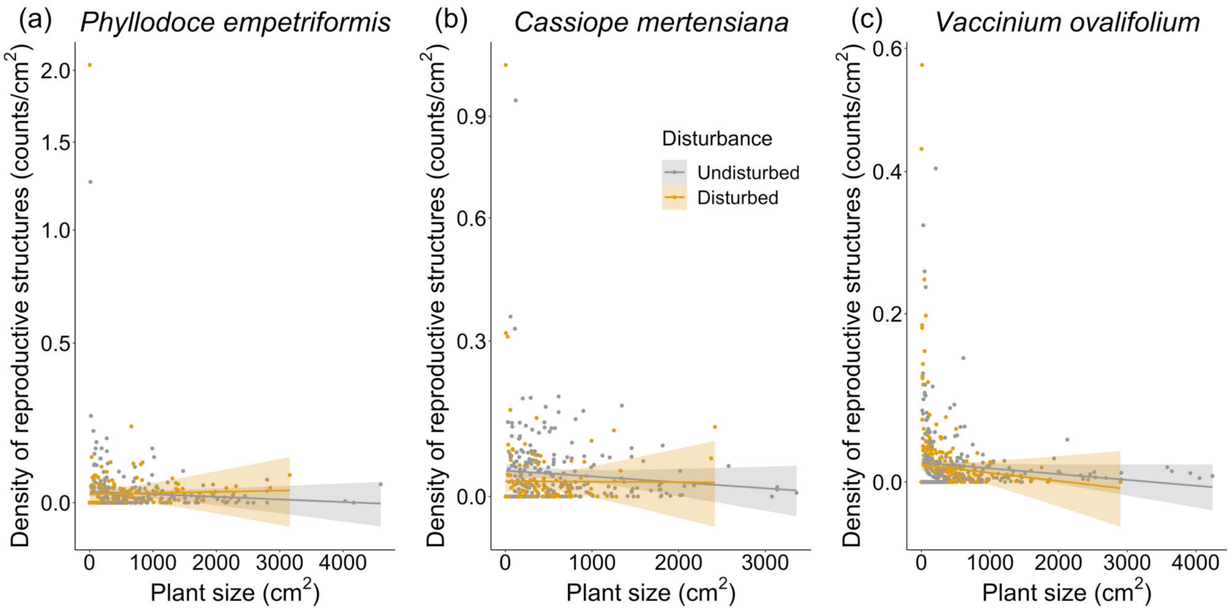

Figure S2. Reproductive structure density. Density of reproductive structures is not dependent on plant size for any of our focal plants (alpha = 0.1). We fit a Linear Mixed Model to test the effects of plant area, disturbance, and their interaction on reproductive density (number of reproductive structures/plant area) for each species in the package 'lmerTest' (Kuznetsova, Brockhoff & Christensen, 2017). We accounted for our nested sampling design with a random intercept for transect pair, and accounted for different responses between species by fitting a separate model for each species (reproductive density ~ plant area * disturbance + (1|transect pair). Shown are confidence intervals (shading) from a linear model and fitted lines are for illustrative purposes only, as they do not account for our hierarchical sampling design. One small *P. empetriformis* plant had 20.4 reproductive structures per cm^2 and this is not visualized in the plot to increase figure clarity. Legend for all plots is as in **(b)**.
